# FAIRness through automation: development of an automated medical data integration infrastructure for FAIR health data in a maximum care university hospital

**DOI:** 10.1186/s12911-023-02195-3

**Published:** 2023-05-15

**Authors:** Marcel Parciak, Markus Suhr, Christian Schmidt, Caroline Bönisch, Benjamin Löhnhardt, Dorothea Kesztyüs, Tibor Kesztyüs

**Affiliations:** 1 Department of Medical Informatics, University Medical Center Göttingen, Von-Siebold-Straße 3, 37075 Göttingen, Germany; 2 University MS Center, Biomedical Research Institute (BIOMED), Hasselt University, Agoralaan Building C, 3590 Diepenbeek, Belgium; 3 Data Science Institute (DSI), Hasselt University, Agoralaan Building D, 3590 Diepenbeek, Belgium; 4 NextLytics AG, Kapellenstrasse 37, 65719 Hofheim Am Taunus, Germany

**Keywords:** Medical data reuse, Electronic health record, Medical data integration center, Automated medical data processing, Medical informatics, Maximum care hospital

## Abstract

**Background:**

Secondary use of routine medical data is key to large-scale clinical and health services research. In a maximum care hospital, the volume of data generated exceeds the limits of big data on a daily basis. This so-called “real world data” are essential to complement knowledge and results from clinical trials. Furthermore, big data may help in establishing precision medicine. However, manual data extraction and annotation workflows to transfer routine data into research data would be complex and inefficient. Generally, best practices for managing research data focus on data output rather than the entire data journey from primary sources to analysis. To eventually make routinely collected data usable and available for research, many hurdles have to be overcome. In this work, we present the implementation of an automated framework for timely processing of clinical care data including free texts and genetic data (non-structured data) and centralized storage as Findable, Accessible, Interoperable, Reusable (FAIR) research data in a maximum care university hospital.

**Methods:**

We identify data processing workflows necessary to operate a medical research data service unit in a maximum care hospital. We decompose structurally equal tasks into elementary sub-processes and propose a framework for general data processing. We base our processes on open-source software-components and, where necessary, custom-built generic tools.

**Results:**

We demonstrate the application of our proposed framework in practice by describing its use in our Medical Data Integration Center (MeDIC). Our microservices-based and fully open-source data processing automation framework incorporates a complete recording of data management and manipulation activities. The prototype implementation also includes a metadata schema for data provenance and a process validation concept. All requirements of a MeDIC are orchestrated within the proposed framework: Data input from many heterogeneous sources, pseudonymization and harmonization, integration in a data warehouse and finally possibilities for extraction or aggregation of data for research purposes according to data protection requirements.

**Conclusion:**

Though the framework is not a panacea for bringing routine-based research data into compliance with FAIR principles, it provides a much-needed possibility to process data in a fully automated, traceable, and reproducible manner.

## Background

Cross-organizational secondary use of medical data is the key to large scale clinical and health services research and essentially important for establishing precision medicine. Reuse of routinely collected data offers extended sample sizes and follow-up times at lower costs and a more representative view of clinical practice in the real-world [1]⁠. The “FAIR Principles for scientific data management and stewardship” were established to make data and the context of their generation Findable, Accessible, Interoperable, and Reusable. These principles summarize common data governance guidelines across multiple research domains with a special emphasis on the automatization of finding and using data [2]⁠. Open data sharing platforms like DataONE or the meta-repositories like DataMed show the applicability of FAIR in real world examples [3, 4]⁠. Both examples enable sharing of research datasets for reuse. However, the data platform presented here, collects and processes data generated during diagnostics and treatment of patients in clinical care at the university hospital and thus sets itself apart from the pre-processed and target-oriented research data, that DataONE and DataMed lean on. In the area of medical research, privacy concerns still remain about the open sharing health data, which oppose the publication of datasets in central repositories [5]. This holds especially true for real world data captured in routine healthcare, which contains patient identifying attributes and requires explicit legal clearing for secondary use in research. In Germany, the Medical Informatics Initiative (MI-I) funds development and implementation of medical data integration centers to create a technical and legal framework for cross-site secondary use of routine healthcare data [6]. As part of the HiGHmed consortium and the MI-I funding scheme, the University Medical Center Göttingen (UMG) implemented such a medical data integration center (UMG-MeDIC) [7]⁠. Establishing data warehousing processes from scratch, we aim for high compliance with the FAIR Principles but face the challenge that data integration workflows are complex and inefficient when done manually [8]⁠. As with any complex software engineering task, documentation is often neglected, not only for software artifacts themselves, but also for any executed workflow [9]⁠. Although data about past workflow runs is highly useful, capturing this type of information is a difficult task [10]⁠.

### Problem statement

**Fig. 1:**
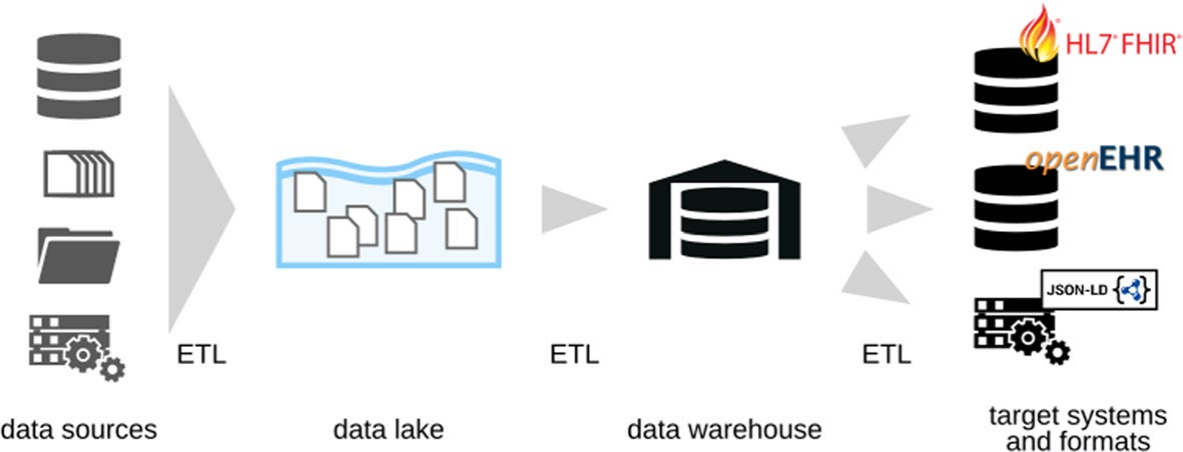
High level view of the logical data flow at the UMG-MeDIC, depicting the different stages of the Extract-Transform-Load process. The information (i.e. healthcare data from the University Medical Center Göttingen) is extracted from the data sources and pooled in a data lake. Within the transformation and loading step the data is pushed to the data warehouse and is then provided to the target systems in the required format. UMG-MeDIC University Medical Center Göttingen-Medical Data Integration Center, ETL Extract-Transform-Load

Sharing research data necessitates an infrastructure that allows data to be found and accessed in an interoperable and reusable format [2]⁠. As real world health data tends to stay in heterogeneous non-FAIR data silos, data engineers need to implement appropriate data integration workflows to make this data available [8, 11]⁠. In contrast to other data-intensive research domains, the healthcare domain imposes additional requirements on data engineering [5, 12, 13]⁠. Moreover, the UMG-MeDIC operates on a continuous flow of data instead of self-contained datasets. Current approaches for FAIR research data management tend to focus on manually "FAIRifying" individual datasets that are published in dedicated research data repositories, neglecting data processing steps prior to obtaining data [14]⁠. Our data integration workflows have to be executed periodically and repeatedly, continuously moving data from multiple source systems to a central data warehouse component to again many target systems. This results in constantly evolving datasets, as illustrated in Fig. 1, that defy manual "FAIRification".

A trustworthy, fully validated data basis, which consists of structured and unstructured data, is therefore the minimum requirement for successful operation. All data processing and management tasks and comprehensive data provenance must be accounted for to enable meaningful scientific application.

Hence, in this article, we describe our implementation approach for a data processing automation framework in a medical data integration center of a maximum care hospital facing the challenges of multi-dimensional data warehousing and processing. Based on this framework, the UMG-MeDIC is dedicated to serve as a research service unit.

## Methods

In order to obtain a comparative overview of proposed approaches from other projects with similar requirements, a comprehensive literature review must first be conducted. The results are then described and discussed in terms of a possible solution to the specific requirements of our MeDIC project.

In a second step, the goals for the implementation of the framework in the MeDIC are defined. Additionally, frequently used concepts and standards are introduced.

The third step identifies workflow sub-processes and corresponding software tools for the realization.

The fourth and last step involves the implementation of the objectives into the framework of the UMG-MeDIC, using the appropriate open-source software components.

### Literature review

PubMed, Embase via Ovid and Web of Science were searched using specific search terms and keywords. The search strategy for PubMed is depicted as an example in Table 1.

**Table 1:** Search history in PubMed

Search Step	Search string	Number of hits
#1	"FAIR principles"[All Fields] OR ("FAIR"[ti] AND "principles"[ti])	103
#2	findab*"[All Fields] AND "access*"[All Fields] AND "interopera*"[All Fields] AND "reusab*"[All Fields]	248
#3	"data warehousing"[MeSH Terms] OR "databas*"[All Fields]	658,390
#4	(#1 OR #2) AND #3	138

In addition to PubMed, the search in Web of Science resulted in 120 hits, the one in Embase delivered 98. Subsequently, a total of 356 references was imported into Refworks and 129 duplicates were removed automatically, leaving 219 references to be screened. Some of the concepts under consideration will be presented in the following.

The FAIR principles have gained a lot of momentum in the research community, resulting in various solutions and proof-of-concepts presenting FAIR data. Initiatives like DataMed and GO FAIR further imply that using Fast Healthcare Interoperability Resources (FHIR) means a central repository component is sufficient to enable persistent accessibility and this architecture would scale for large data volume [4, 12]⁠. Usage of semantic modeling languages like FHIR or openEHR may contribute to overall FAIRness and especially reusability of data [15]⁠. Contrary to the usage of FHIR within GO FAIR or DataMed, the UMG-MeDIC incorporates a data warehouse as central repository component, using a Structured Query Language (SQL)-based database, to store pseudonymized data. FHIR is, in the environment of the UMG-MeDIC, rather intended as an exchange format, than a central repository component.

The Emergency Department Catalog (EDCat) system was developed to improve the FAIRness of a project considering emergency department databases but still requires manual organization of datasets, which, in view of the volume of data, is not suitable for the operation of a MeDIC and further pursuit of this solution was discarded [14]⁠.

The SCALEUS-FD offers a semantic web tool that allows data integration and reuse in compliance with FAIR Data principles and was validated in the domain of rare diseases, where records are rather small, not comparable to the volume of data that has to be processed in a maximum-care hospital each day [16]⁠. Since there was no experience with large numbers of data points and records in the SCALEUS-FD, but the way to handle big data was mandatory for the UMG-MeDIC, this approach was not considered further.

On the contrary, the YOUth cohort study, a large-scale longitudinal cohort study with highly sensitive data, faced similar requirements concerning privacy, heterogeneous data and sources, and data quality checks [17]⁠. The most important and decisive difference, however, lies in the way and objective of the data collection. While the data of the well-defined cohort are obtained a priori for research purposes, standardized and per protocol, the data of a MeDIC are primarily routinely collected, often also referred to as “real world data” [18]. The implicit difference in standardization and quality requires a correspondingly differentiated and more elaborate data management in order to finally make the latter usable for research.

The European Medical Information Framework (EMIF) created a catalog of data sources from research studies and routine clinical care to enable researchers to find, access, and reuse datasets while respecting privacy [19]⁠. For this purpose, a four-layer concept was developed in which each layer can be authorized individually, thus enabling different degrees of data access. However, unlike the MeDIC, they do not primarily aim to integrate the data itself, but rather consolidate various data resources into an overarching biomedical marketplace based on FAIR principles [20]⁠.

In the biomedical environment, a vast amount of data for processing, in other words big data, concerns not only but above all omics-data. Hence, the tools and solutions applied can be similar to our concept, with the crucial difference that the diversity of data types is generally low in omics-data and very high in a maximum-care university hospital and therefore in the UMG-MeDIC.

In summary, there are many examples from different fields that aim to provide data according to FAIR principles to enable further research and offer the best possible patient-centered care or precision medicine. However, we could not find a solution that meets all our challenges of different data types, large amounts of data, high-velocity and timeliness, and processing of structured and non-structured clinical care data.

### Goals

As no appropriate pre-existing solution could be identified, as portrayed in the prior subsection, we iteratively implement a prototype based on best of breed open source components. Primary goals for our implementation of a trusted medical research data integration system were:

- Operate an integrated data warehouse
- Ingest data from any number of source systems in any data format in batch or near real-time schedule
- Provide data into a varying number of target systems in data format
- Ability to scale data processing flexibly
- Orchestrate all data processing tasks across network security zone barriers
- Ability to monitor status and history of all data processing tasks
- Operate the entire system in a high level information security context with a special focus on data integrity and (long term) accessibility

In addition to the software used, which will be identified and presented in the next step, frequently used standards and concepts are introduced at this point for a better understanding of the approach. Table 2 provides an overview of these standards and concepts with a brief explanation of each.

**Table 2:** Standards and concepts used in the development of the UMG-MeDIC framework

Term / acronym	Resolved	Short description
ACID	Atomic Consistent Isolated Durable	A set of standard properties that ensure reliable processing of database transactions
CSV	Comma Separated Values	Structure of a text file for storage or exchange of simply patterned data
Data lake		System or repository of structured or unstructured data, including raw copies of source system data and transformed data
DWH	Data warehouse	A central database optimized for analysis purposes that combines data from several, usually heterogeneous sources
DRG	Diagnoses Related Groups	Diagnosis-related grouping of patient cases with similar costs, used for medical billing
ETL	Extract Transform Load	An integrative process in which data is extracted from multiple sources, which may have different structures, processed, and merged into a target database
FAIR	Findable Accessible Interoperable Reusable	The principles were defined in 2016 by a consortium of scientists and organizations and emphasize machine-actionability with regard to the increase in volume, complexity, and creation speed of data
FHIR	Fast Healthcare Interoperability Resources	Fast Healthcare Interoperability Resources is a standard developed by HL7 that supports data exchange between healthcare software systems
HL7	Health Level 7	Health Level 7 is a non-profit, ANSI-accredited organization developing standards for the exchange of information between healthcare services
HTTP	Hypertext Transfer Protocol	Regulates the communication between browser and web server
IDE	Integrated Development Environment	Application development software that combines common developer tools in a central graphical interface
IRIs	Internationalized Resource Identifiers	internationalized form of the Uniform Resource Identifier (URI), an identifier consisting of a string of characters used to identify an abstract or physical resource
open EHR	open Electronic Health Record	An open standard health informatics specification for managing, storing, retrieving, and exchanging health data in electronic health records
LOINC	Logical Observation Identifiers Names and Codes	international standard of universally accepted names and identifiers of health measurements, observations and documents
REST API	Representational State Transfer Application Programming Interface	REST APIs communicate via HTTP requests to perform standard database functions such as creating, reading, updating, and deleting records within a resource
SQL	Structured Query Language	SQL is a database language for defining data structures and for editing and querying datasets in relational databases
TLS	Transport Layer Security	Encryption protocol for secure data transmission on the Internet
URL	Uniform Resource Locator	Identifies and locates a resource via the access method to be used and the location of the resource, e.g. web page via HTTP

### Identification of workflow sub-processes and corresponding software tools

**Fig. 2:**
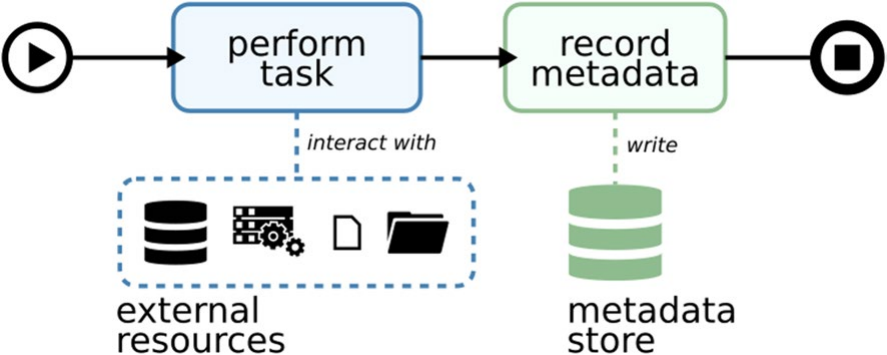
Generic schema of an atomic Extract Transform Load task and metadata capture sub process. Process control flow: black lines left to right; data flow: blue outline; metadata flow: green outline. The process is started with a “perform task” which interacts with external resources and pulls data from different sources. Subsequently the process flow enables the recording of metadata that is written in a separate metadata storage

The required data integration workflows for operating the UMG-MeDIC (according to the goals defined above) can be divided into sub-processes and strategically split into modules, which are equal for many workflows. In our bottom-up description of the sub-processes, we start with the atomic data processing tasks, which can be summarized as follows: A data processing task takes input data and manipulates it in a well-defined manner to produce output data whenever triggered by the process control flow. Each processing task must be documented with process metadata containing all necessary information to recreate the exact parameters of its execution as well as the relevant runtime environment. The full process control flow includes the actual data processing task and the recording of process metadata (see Fig. 2). These atomic processing tasks may be concatenated by the control flow into an end-to-end data processing workflow that moves information across multiple storage systems and formats.

Based on the aforementioned sub-process tasks, we had to take into account an agnostic implementation of this fundamental framework. This includes the programming language or software tool used to implement actual data processing tasks, and information model used to document process metadata to be manufacturer-independent. We have chosen the following components: An orchestration system that manages process control flow, a compatible task execution engine, and a semantic model, storage service and documentation engine for process metadata.

For our prototype implementation, we selected a set of tools published under open source licenses, described in Table 3, to implement these components.

**Table 3:** Overview of tools implemented to perform the tasks of the UMG-MeDIC

**Process Metadata**				
Software	**JSON-LD**	**Schema.org**	**CouchDB**	
Description	A serialization technique for linked data (LD) objects using the JavaScript Object Notation (JSON) [21, 22]⁠. This technique allows assigning unique identifiers using Internationalized Resource Identifiers (IRIs) for JSON objects and consequently to use these identifiers as references [23]⁠	The collaborative and hierarchical vocabulary allows to create semantically standardized and linked metadata information [24]⁠. It is serializable in different formats including JSON-LD. All elements from Schema.org are described in detail, allowing to define metadata understandable by humans and machines alike. Predefined types like Schema.org Dataset or DataDownload summarize relevant metadata elements that can be attached to data from the UMG-MeDIC [24]⁠	Apache Cluster of unreliable commodity hardware Data Base (CouchDB) is a JSON based document database [25]⁠. Data can be read, written, modified, or deleted using a Representational State Transfer Application Programming Interface (REST API). Built for large deployments, CouchDB allows to be quickly replicated to multiple servers while maintaining the ACID (atomic, consistent, isolated, durable) properties of the database. Solution to store JSON-LD process metadata documents	
**Process Flow**				
Software	**ActiveWorkflow**	**Docker**	**Celery**	**Data Storage CDSTAR**
Description	A web-based automation engine to orchestrate and monitor workflows [26]. A web-GUI allows to define and run a workflow consisting of individual agents. Each agent is an autonomous software service that communicates with the workflow engine via a standardized REST API. Workflows can be executed event based or on a predefined schedule	A runtime for software containers [27]⁠. A software container is a lightweight and interoperable application bundle. These bundles include all requirements and can be run by the Docker engine, which manages networking, data storage and monitoring of run containers. Running containers with Docker is a lightweight, software-defined alternative to server virtualization. Runtime environment templates called Docker images enable fully reproducible process execution as well as encapsulation and preservation of the entire virtualized runtime environment	Highly scalable distributed message queue for task scheduling [28]⁠. It works message-based, brokering messages to worker nodes and collecting results into a backend. Used to asynchronously execute atomic data processing tasks	Common Data Storage Architecture (CDSTAR) is a data storage abstraction layer [29]. It abstracts physical storage solutions and offers storing, reading and modifying data via a REST API. CDSTAR is organized into vaults, for which individual authorization can be set. A vault holds archives, uniquely identified by an archive ID. An archive contains individual files, identified in turn by internal ID or a filename. CDSTAR is used as a lightweight data lake solution

## Results

In this section, the implementation of our framework and the inherent workflow are described and an example is given to illustrate the function of the framework.

Currently, the following hospital department systems are connected to the UMG-MeDIC: Laboratory system (Opus::L), administration system (SAP: transaction data, billing data), microbiology and virology system (MLAB), clinical tumor documentation (Onkostar), cardiology system (CCW, including echocardiography, cardiac catheterization), sensor data intensive care (ICCA), medication and substances (Meona), and emergency admission (E.Care). Soon to follow will be clinical trial software (secuTrial), image data (PACS), the pathology system (Nexus/IMS), surgical data (Win-OP), radiology (structured findings), treatment quality data (QS-Med), and dental data. Since the UMG-MeDIC is still in the process of being set up, not all departmental systems are connected yet. However, these will all follow and thus contribute the full range of data to be expected in a maximum care university hospital.

### Overview

**Fig. 3:**
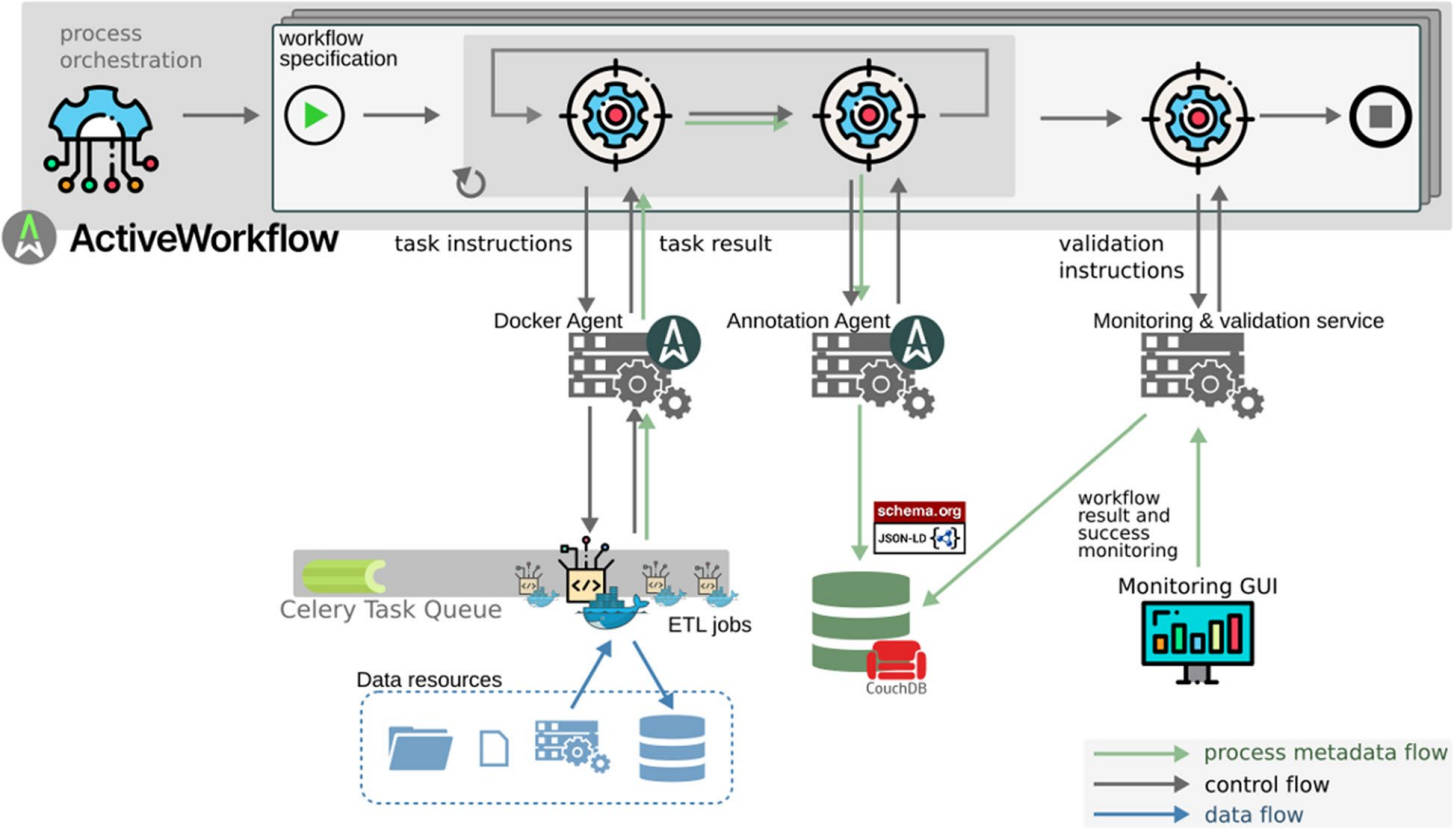
System architecture of the implementation. The complete process is, as described in the text, divided in tasks, which are controlled by the ActiveWorkflow system. ETL Extract-Transform-Load, GUI Graphical User interface

The UMG-MeDIC infrastructure follows the microservice paradigm. We operate each application as an autonomous service. ActiveWorkflow orchestrates process flows along these micro-services with workflow specifications being defined by UMG-MeDIC data engineers. Based on a given workflow definition, ActiveWorkflow communicates with the other services through so-called agents. An agent implements the ActiveWorkflow REST API and works autonomously and asynchronously of ActiveWorkflow itself. ActiveWorkflow communicates with the agents using JSON document in the HTTP message body. A JSON message can hold any (text-based) payload. Figure 3 shows an overview of the service ecosystem used to implement our task automation framework.

Against the background of the volume of data to be handled daily we aimed to automate the workflows for managing all kind of incoming clinical care data and the subsequent curating and provision processes of research data. Metadata about this automated processing have to be captured, collected, and published to enable traceability and reproducibility of data transformation as well as the publication of detailed data provenance records. By using JSON-LD metadata templates, we capture relevant information and create linked data compatible with provenance information models. Our implementation uses containerization to allow platform-agnostic execution and reproduction of any data integration workflow. We employ data lake web services to persist copies of all incoming source and intermediate data artifacts. All components are autonomous and communicate through RESTful web service application programming interfaces (API), allowing to be operated in compliance with strong IT-security policies.

### Metadata processing

We implemented a number of custom ActiveWorkflow agents to assist our data integration workflows. The Docker Agent allows executing generic Docker, and in our case, these Docker images are usually Extract Transform Load (ETL) jobs implemented in Python. The Annotation Agent collects process metadata and writes this data to our CouchDB process metadata store, which contains JSON-LD documents based on Schema.org metadata templates. These are two types of documents: metadata regarding datasets used as input or produced by ETL jobs, and metadata concerning the processes that manipulated a dataset. Figure 3 depicts all flows of metadata as green arrows.

The sequence of Docker Agent and Annotation Agent tasks in a workflow specification can be repeated as necessary until all logical steps of a desired ETL pipeline are completed. As a simple example, the "extract" part could be split from the "transform" and "load" parts of a pipeline to first store a persistent copy of the source data before applying further manipulation. A full example of data processing pipelines at UMG-MeDIC is described below.

#### Templates

We defined metadata templates in order to standardize capturing of relevant information during data integration workflows. Schema.org definitions for Dataset and DataDownload provide the basis for archives and files of our CDSTAR data lake, respectively [24]⁠. Modeled after CDSTAR, a Dataset document has multiple parts (hasPart) of DataDownload documents. The inverse property isPartOf pointing from DataDownloads to a Dataset does also hold. Both metadata documents link to their CDSTAR counterparts. The data contained in the metadata documents consists of two parts: a redundant copy of the metadata provided by CDSTAR as well as manual descriptions written by the data engineer responsible for a data integration workflow. The manual descriptions are added through the workflow definition in ActiveWorkflow to every dataset processed.

Processes are modeled as CreateAction documents. These documents contain an object and a result reference element to indicate input and output data respectively. An instrument element references an implemented data integration processing step represented as a SoftwareApplication. The SoftwareApplication object again references the source code repository in GitLab as “downloadUrl” to uniquely identify the code that ran. Moreover, supportingData refers to any configuration variables that may be supplied to the ETL process implementation influencing the code execution. Finally, each process metadata document contains an isPartOf reference to the workflow description. The workflow description is manually created as human-readable process documentation. We support this manual documentation step by exporting the workflow definition from ActiveWorkflow and enriching it with free-text descriptions.

### Workflow monitoring and validation

**Fig. 4:**
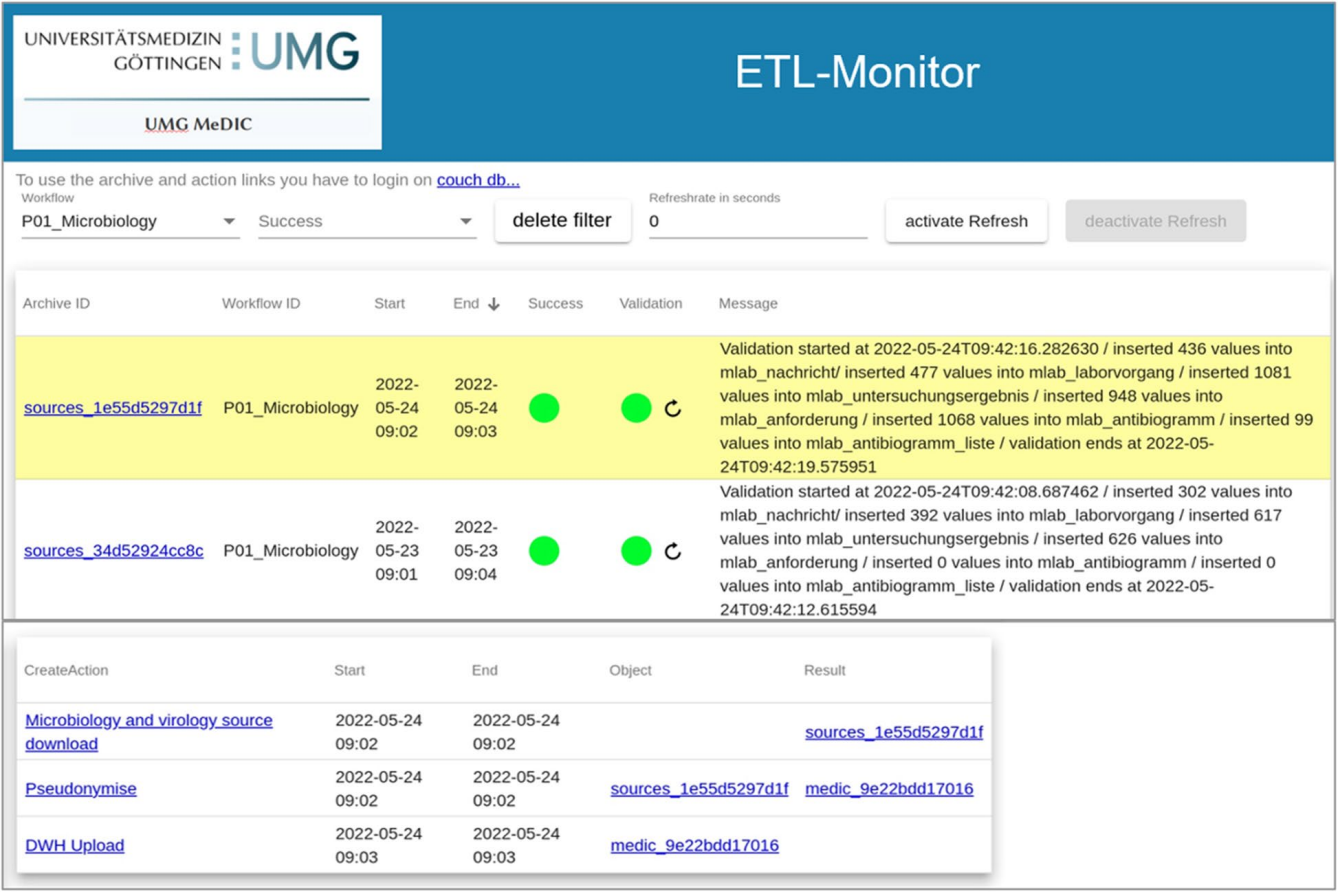
ETL-Monitor: Extract-Transform-Load-processes are displayed with their respective status (success, fail). Here, for example, the work flow of importing microbiology data is displayed. Clicking on a specific process provides detailed information. If the process failed, the last step that was successful is displayed. UMG-MeDIC University Medical Center Göttingen-Medical Data Integration Center, DWH Data Warehouse

We implemented a custom monitoring service and web-based user interface based on the collected process metadata. The monitoring service extracts process metadata documents from CouchDB and displays it in the interface from a workflow perspective. All processing steps that belong to a specific run of any defined workflow are displayed in sequential order. If any workflow run fails to reach its successful final state, a visual warning is displayed to the user. Figure 4 shows the ETL-Monitor running at the UMG MeDIC. To validate correct execution of each workflow run, a second status indicator is presented based on the actual data that was processed. During workflow execution, statistical parameters of the processed data are read and captured as part of the process metadata documents. At the end of each workflow run, a validation service checks whether the same statistical parameters can be re-calculated on the data loaded into the target system. Since data format, content, and expected transformations differ for each source or target system, custom validation functions are implemented per workflow. We start with a set of simple methods, like row counts, and develop more complex functions over time as needed. Validation results are again stored as part of the process metadata in CouchDB.

#### Example workflow

To better illustrate how the proposed data processing framework translates into practical application, we describe an exemplary workflow in more detail. Data storage systems, the type of information to process, and the methods to achieve processing are subjective choices that fit our specific situation. The framework can also be applied with entirely different implementation choices.

**Fig. 5:**
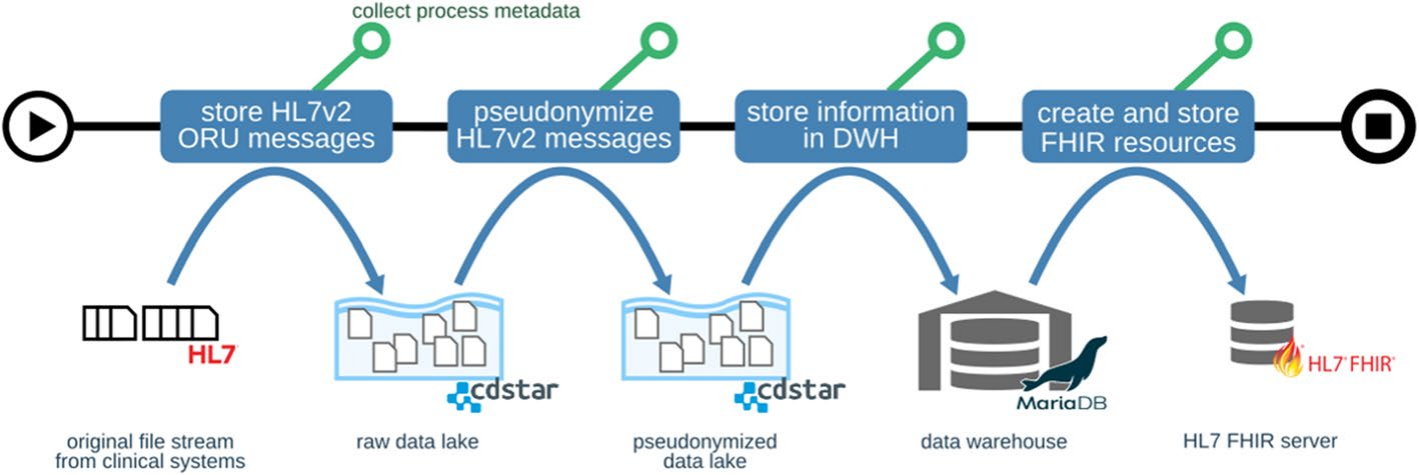
Schematic representation of data flow between different storage systems. The example workflow shows transfer of laboratory result information through common stages of data processing and storage at UMG-MeDIC. The process starts with the HL7 file stream from the clinical systems, where the observation results are stored in a ORU message and the corresponding process metadata is collected. The information is pooled into a raw data lake. Subsequently the information is pseudonymized and transferred in a pseudonymized data lake. After preprocessing the information is stored in the data warehouse. In a final step FHIR resources based on the data are created and stored in a HL7 FHIR server. UMG-MeDIC University Medical Center Göttingen-Medical Data Integration Center, HL7 Health Level 7, ORU HL7 Observation Result, FHIR Fast Healthcare Interoperability Resources, DWH Data Warehouse

The logic of the example workflow is as follows: laboratory results enriched with LOINC codes for all patients treated at the UMG are communicated to all hospital department systems in HL7 Version 2 standard "ORU" (HL7 Observation Result) messages, via a clinical communication server. The UMG-MeDIC is registered as a receiver of this message stream, which is the primary input for the workflow. A HL7 message contains identification data (IDAT) of the patient, like the patient id, the name and the date of birth. Additionally, the HL7 message includes the medical data (MDAT) such as lab values, LOINC codes etc. Information from these messages is to be extracted and pseudonymized in a process, where the IDAT is extracted, and a unique number is created by a special algorithm. The personal data, such as the name, is deleted and only the year is stored from the date of birth. This step replaces the IDAT with the pseudonym data (PDAT). The complete pseudonymization process takes place in a protected network segment called "patlan". After pseudonymization, the MDAT connected to the PDAT is ready to be transferred to the "medic" network segment. The information of the message is then transformed to a relational data model and stored in the central data warehouse system. From the relational database system the information is again extracted and transformed into a HL7 FHIR standard "Observation" resource (representing diagnostic and clinical data), and finally stored in a FHIR Server. Resources in the FHIR Server can ultimately be used for cross-organizational querying of medical data for research purposes within MI-I projects. Figure 5 is a graphical representation of this data flow logic. Each processing and annotation step is implemented using the framework described above.

We employ two distinct data lake instances based on CDSTAR as persistent object storage for all data integration processes. It assigns each dataset a unique identifier, the ArchiveID. Any agent is able to use the combination of CDSTAR URL and ArchiveID to identify and download any dataset in our data lake. CDSTAR enables versioning of datasets. Each modification of any dataset will result in a new version. In Figs. 3 and 5, flow of data artifacts is shown as blue arrows. Two instances are used to split raw data containing patient identifying information and pseudonymized data into different network security zones as required by the UMG-MeDIC information security policy.

We use MariaDB as a curated relational data warehouse. The data warehouse contains pseudonymized and transformed medical datasets. Relational database schema and table definition are defined in consultation of the respective source data custodians. The schema aims to cover as much information as possible provided from the sources and enables integrated queries across data from all sources. In addition, all information present in the data warehouse contains an attribute ArchiveID. The ArchiveID refers back to a dataset in the data lake, indicating the origin of any data in the data warehouse.

The exemplary lab result workflow concludes with an ETL step that extracts information from the data warehouse, creates HL7 FHIR Observation resources, and stores these in a FHIR server. This step is an example of how integrated data at UMG-MeDIC may be curated for researcher access and offered in different formats, according to the specific requirements of the research project. Transformation to FHIR format can be replaced with any given semantic data model, e.g. openEHR or custom CSV. These output formats vary and will be regularly extended by the data engineering team. The generic nature of our data processing framework supports frequent addition of new output data pipelines.

#### Example ETL-process

**Fig. 6:**
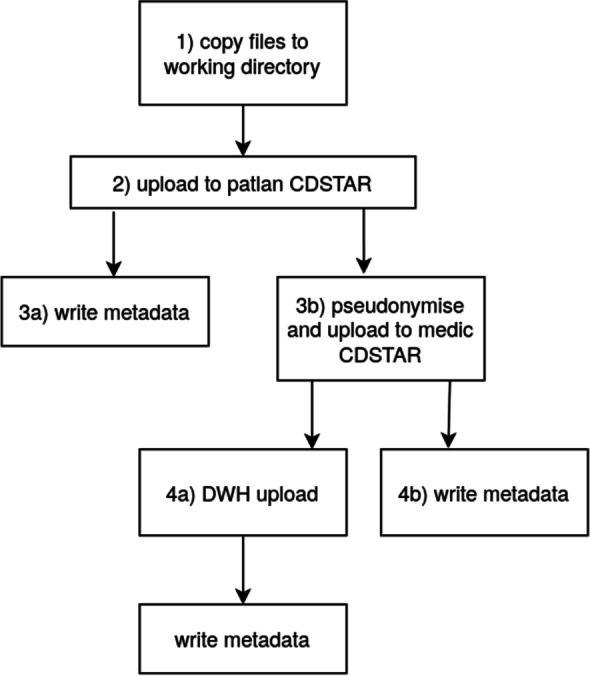
Graphical representation of a standard ETL Workflow. ETL Extract-Transform-Load, CDSTAR Common Data Storage Architecture

Figure 6 illustrates the exemplary ETL-workflow of microbiology data from the source system (MLAB) to the DWH in several steps. First, the MLAB files are copied from the mount-folder to a working directory (1), then the files are loaded into the patlan CDSTAR (2) and metadata concerning (2) is written to CouchDB (3a). The HL7 file is pseudonymized (3b) and subsequently parsed, and the information it contains is inserted into a series of tables in the DWH (4a) and respective metadata are written to CochDB (4b).

These inserts include the patient and case number information stored in the HL7 file, the particular laboratory tests requested and their findings, and the sample material. The latter refers, for instance, to the body region from which the material was taken, and enables the storage of information on multiple samples that may occur in the HL7 files. Furthermore, the result of each examination and a reference to the corresponding laboratory test are stored. Antibiograms pertaining to the possible resistance of bacteria to antibiotics are also stored in separate tables to support multiple antibiograms with various bacteria and different antibiotics. Finally, metadata is written to CouchDB regarding the DWH-upload (5).

## Discussion

### Implementation of the framework

The proposed data processing automation framework meets the requirements for data management tasks and contextual constraints of the UMG-MeDIC as defined above. We successfully implemented the framework and operate on real-world data from many source systems. Source data is persistently stored in data lake services and transferred into an integrated relational data warehouse. Information from the data warehouse is collected into different subsets, transformed and stored into target systems for research use cases such as the Medical Informatics-Initiative or the HiGHmed project. The components are divided into multiple network security zones as required by the UMG-MeDIC information security policy. Communication across network zones is allowed along well-defined unidirectional HTTP routes while still enabling full workflow control through a single ActiveWorkflow GUI for the data engineering team. Capability for data processing can be increased by horizontally scaling the Celery task queue to include more compute nodes if necessary. Monitoring of workflow progression and success is implemented based on automatically captured process metadata and enables quick status checks for data engineers in day-to-day operation of many automated workflows. Maintaining long-term integrity and availability of the handled data is a challenge for the operational processes and therefore outside the scope of the proposed framework. The choices to permanently store input and intermediate datasets in data lake services and tracking all process metadata are fundamental in enabling long-term preservation, routine integrity checks, and (public) availability of metadata.

Data flow and metadata annotation are implemented as independent yet interwoven subsystems of micro-services. It is possible to extend or exchange one subsystem without having to discard the other. This means that the information model of metadata capture or the storage engine may be altered at any point in time to reflect consolidation of domain or global standards. The same applies for the data lake storage layer, which is open for addition of further services, i.e. scaling out to (on-site) object storage cluster if needed. Data processing steps are encapsulated within Docker images, which again allows for a high degree of flexibility as data engineers are not forced to implement the required functionality in a given programming language but may choose whatever tooling fits the use case best. The system is open to the integration of any legacy transformation processes, which may be packaged and run as a Docker container. Finally, the orchestration engine itself is interchangeable. The concept is in principle open to be orchestrated by any controlling mechanism that is able to subsequently call RESTful web-services and pass data from one to the next. We chose ActiveWorkflow as our orchestration engine over larger open source projects like Apache Airflow or Luigi mainly because of accessibility and the fact that the plugin system is REST API based and thus again independent of any specific programming language.

### Challenges and limitations

The high degree of flexibility described above is a double-edged sword. If the system architecture is so generic in principle, the people developing, operating, and maintaining the system need to have a clear understanding of common goals and tools. A strict set of guidelines must be agreed upon and followed to avoid unnecessary creolization of implementation methods and expansion of complexity. In our case, we decided to consolidate all programming efforts on Python. Legacy ETL jobs designed and implemented with Talend Open Studio for Data Integration have been incorporated to avoid premature refactoring. The data processing workflows themselves follow a consolidated structure, reducing implementation and documentation effort within the data engineering team.

One drawback to the REST-based extensibility of ActiveWorkflow is that there are no advanced methods to secure its REST service endpoints. The ActiveWorkflow Remote Agent protocol does not yet support authentication mechanisms like HTTP Basic Authentication or Token based methods like OAuth. Our custom Remote Agents are implemented with an "API key" parameter that must be specified in the agent configuration in the ActiveWorkflow user interface. Since that parameter is transmitted in plain text as part of the HTTP message body, encrypted TLS communication should always be used between ActiveWorkflow controller and Remote Agent services. This circumstance is not problematic for our setup, as all instances of the services are operated in dedicated network zones shielded against external requests or attacks. Exposing a Remote Agent service to the public internet is not recommended at the moment. Due to our custom Docker Agent connecting to the host server's Docker engine, it must be well protected and workflow admins need to be aware of the security implications of executing arbitrary images.

### Lessons learned

During implementation of the framework, a major lesson learned was to embrace actual programming languages when developing ETL jobs, in our case Python. The internal best practice had been Talend Open Studio for Data Integration, a fully graphical IDE that allows users to create and execute no-code (or at least low-code) ETL jobs. While this tool had been successfully used for data integration processes in various research projects, we quickly hit walls when scaling across multiple users, network security zones, and the multitude of pipelines required for the UMG-MeDIC. Source code and the software engineering tool-chain are mature with regard to accessibility, versioning, multi-user interaction, review and refactoring workflows, change management and documentation processes, and are common knowledge among employees and candidates from the software engineering domain. We have experienced a major increase of transparency and productivity of the data engineering team since selecting Python and its data science libraries as primary ETL development tools.

### Data warehouse architecture

Considering data warehousing architecture patterns, Armbrust et al. propose a third generation architecture that completely removes a curated relational data warehouse and performs all operation directly on a data lake component [30]⁠. We have explicitly decided to opt for a second generation architecture that combines data lake component for ingested datasets and intermediate artifacts but still emphasizes the curated warehouse as the core component for data integration and semantic enrichment. Where required by technical constraints of the use cases, datasets will be directly pulled from data lake components into analysis processes, e.g. where medical imaging artifacts contain the relevant information. In these use cases, we pull relevant metadata about the available raw data items into our data warehouse and enable stratified search for source data collections based on the integrated medical facts from all clinical department systems indexed at UMG-MeDIC. As a dedicated service unit of a large university hospital, we do simply not face the challenges of scale that lead to the development of a third generation architecture. Benefits of a tightly curated model and expertise about all content indexed at the data warehouse are far more important for our primary goal of guaranteed quality of the information provided to our research partners.

### Ongoing processes of further development

We consider our current data processing automation framework and its implementation an ongoing work, because of changing requirements that arise from the ongoing development in the clinical routine. Requirements change over time, new functionality might become necessary with new data sources or data formats we encounter. The modular nature of the framework will be key to constantly extend and refine components. One major area of expansion in the coming years will be metadata cataloging and governance. The current process of metadata capturing is merely the tip of the iceberg. Metadata about medical facts, as well as metadata about source system state, consent and access rights, schema, format, medical vocabulary and mapped ontology terms is already collected in certain places and retrieved by the systems. Here, however, an expansion of the information must already take place at the source system. The list can be almost indefinitely be continued. To document all these types of metadata and to link their semantic meaning back to the actual data items opens up a new load of functional requirements. The user base also extends beyond just the data engineering team and will include domain experts, data stewards, and project managers. While some of these metadata-related challenges are exclusive to medical data, a lot may be learned and repurposed from the existing and growing ecosystem of open source big data metadata management tools like the Egeria Project or Amundsen [31, 32].

Our process metadata schema will most definitely be adjust in the future as the field of data provenance research enters a consolidation phase [10]⁠. Recent open source developments like the OpenLineage specification and its reference implementation, Marquez, do not yet provide harmonized methods for the full depth of process metadata we collect, but could become viable options down the line [33, 34]⁠. Ultimately, process metadata should be publicly available for datasets that are used in published scientific works and the use of standards for provenance documentation will increase re-usability and value of the information [2]⁠. The push for harmonized data provenance frameworks from the big data community outside academia implied by these recent developments might be an indicator that the idea of linked FAIR data objects reaches a stage where industry adoption increase maturity and usability of prototypes and tools from the scientific community [35, 36]⁠.

The current monitoring and validation mechanisms build into our framework fulfill the basic requirements but might see in-depth refactoring in later iterations. While both are generic and extendable in nature, adaptation of converging solutions from the open source community would be preferable in the end. The topics observability and metrics are gaining traction and fitting open specifications for information models and services are probably already emerging. Validation of data ingest, data in the warehouse, and data produced for specific research use cases is a necessity for a meaningful use of the UMG-MeDIC core services. Current validation only scratches the surface by proving correct behavior of internal data processing. Full content validation would require methods to compare information stored in the original clinical source systems with the information present in the data warehouse, as demonstrated by Denney et al. [37]⁠. Data quality monitoring, development of domain specific data quality metrics and scores are other highly interesting topics that research from UMG-MeDIC will focus on in the future.

With the medical research data warehouse and the data processing framework fully established, focus for subsequent work shifts towards data publication and analysis. To fully comply with the FAIR guiding principles, the process for automatic registration of persistent identifiers and publication of metadata needs to be implemented. Automatic deployment of research data marts as proposed by Spengler et al. hold great potential to generate value for medical researchers with minimal barriers [38]⁠. A comprehensive metadata management system as described above will enable on-the-fly data mart deployment as well as increasingly automatic deployment of ETL pipelines. Once data formats of input and output systems are modeled precisely in a metadata management system, generating code snippets or fully functional workflow definitions may again enable significant gains in efficiency for the data engineering team.

## Conclusions

We analyzed the basic requirements for data processing at a medical research data service unit and proposed a generic architecture framework and prototype implementation that focuses on scalable automation of such tasks. Data extraction from many and heterogeneous sources, including structured and unstructured data, pseudonymization and harmonization, integration and aggregation can be orchestrated completely independent of data and metadata formats. Our implementation works with a custom database schema for initial data harmonization and incorporates Schema.org metadata information model to track data provenance. While highly powerful, extensible, and flexible by design, the prototype implementation is tailored towards operation in a secure internal network environment by privileged users and does not yet incorporate advanced measures to enforce information security in the wild. The framework itself—like any software available today—is not a silver bullet to make research data comply with the FAIR principles but provides much needed ability to process data in a fully automated, traceable, and reproducible manner. If applied comprehensively, the complexity of research data annotation can be largely transferred from the individual researcher into infrastructure. Quality and speed of the data acquisition process that drives scientific insight can be increased.

## Data Availability

All presented software components are freely available on the Internet.

## Abbreviations

ACID: Atomic, consistent, isolated, durable
CDSTAR: Common Data Storage Architecture
CSV: Comma separated values
DRG: Diagnoses Related Groups
DWH: Data Warehouse
EDCat: The Emergency Department Catalog
EMIF: European Medical Information Framework
ENCODE DNase: Encyclopedia of DNA Elements
EHR: Electronic Health Record
ETL: Extract Transform Load
FAIR: Findable, Accessible, Interoperable, Reusable
HL7: Health Level 7
HL7 FHIR: Fast Healthcare Interoperability Resources
HTTP: Hypertext Transfer Protocol
IDE: Integrated development environment
IRIs: Internationalized Resource Identifiers
LD: Linked data
LOINC: Logical Observation Identifiers Names and Codes
ORU: HL7 Observation Result
REST API: Representational State Transfer Application Programming Interface
SQL: Structured Query Language
TLS: Transport Layer Security
UMG-MeDIC: University Medical Center Göttingen (UMG) Medical Data Integration Center
URL: Uniform Resource Locator
